# A safer and more practical tracheotomy in invasive mechanical ventilated patients with COVID-19: A quality improvement study

**DOI:** 10.3389/fsurg.2022.1018637

**Published:** 2022-10-28

**Authors:** Kai Kang, Junfeng Wang, Xue Du, Nana Li, Songgen Jin, Yuanyuan Ji, Xinjia Liu, Pengfei Chen, Chuangshi Yue, Jihan Wu, Xintong Wang, Yujia Tang, Qiqi Lai, Baitao Lu, Yang Gao, Kaijiang Yu

**Affiliations:** ^1^ Department of Critical Care Medicine, The First Affiliated Hospital of Harbin Medical University, Harbin, China; ^2^ Department of Ultrasound, The First Affiliated Hospital of Harbin Medical University, Harbin, China; ^3^ Department of Critical Care Medicine, The Sixth Affiliated Hospital of Harbin Medical University, Harbin, China; ^4^ Institute of Critical Care Medicine, The Sino Russian Medical Research Center of Harbin Medical University, Harbin, China; ^5^ Key Laboratory of Hepatosplenic Surgery, Ministry of Education, Harbin, China; ^6^ Key Laboratory of Cell Transplantation, National Health Commission, Harbin, China

**Keywords:** percutaneous dilated tracheotomy, bedside ultrasonography, delayed endotracheal intubation withdrawal, COVID-19, invasive mechanical ventilation, pre-surgical positioning, intraoperative damage of the posterior tracheal wall, tracheoesophageal fistula

## Abstract

**Importance:**

The number of infections and deaths caused by the global epidemic of severe acute respiratory syndrome coronavirus type 2 (SARS-CoV-2) invasion is steadily increasing daily. In the early stages of outbreak, approximately 15%–20% of patients with coronavirus disease 2019 (COVID-19) inevitably developed severe and critically ill forms of the disease, especially elderly patients and those with several or serious comorbidities. These more severe forms of disease mainly manifest as dyspnea, reduced blood oxygen saturation, severe pneumonia, acute respiratory distress syndrome (ARDS), thus requiring prolonged advanced respiratory support, including high-flow nasal cannula (HFNC), non-invasive mechanical ventilation (NIMV), and invasive mechanical ventilation (IMV).

**Objective:**

This study aimed to propose a safer and more practical tracheotomy in invasive mechanical ventilated patients with COVID-19.

**Design:**

This is a single center quality improvement study.

**Participants:**

Tracheotomy is a necessary and important step in airway management for COVID-19 patients with prolonged endotracheal intubation, IMV, failed extubation, and ventilator dependence. Standardized third-level protection measures and bulky personal protective equipment (PPE) may hugely impede the implementation of tracheotomy, especially when determining the optimal pre-surgical positioning for COVID-19 patients with ambiguous surface position, obesity, short neck or limited neck extension, due to vision impairment, reduced tactile sensation and motility associated with PPE. Consequently, the aim of this study was to propose a safer and more practical tracheotomy, namely percutaneous dilated tracheotomy (PDT) with delayed endotracheal intubation withdrawal under the guidance of bedside ultrasonography without the conventional use of flexible fiberoptic bronchoscopy (FFB), which can accurately determine the optimal pre-surgical positioning, as well as avoid intraoperative damage of the posterior tracheal wall and prevent the occurrence of tracheoesophageal fistula (TEF).

## Background

Coronavirus disease 2019 (COVID-19) is wreaking havoc around the world, with the mutations of severe acute respiratory syndrome coronavirus type 2 (SARS-CoV-2) aggravating the current situation even more (1). The effective spread of SARS-CoV-2 mainly occurs *via* virus-containing respiratory droplets or aerosols, virus-contaminated hands, or surfaces (2–4). In the early stages of outbreak, approximately 15%–20% of patients with COVID-19 inevitably progressed to more severe and critically ill cases especially elderly patients or those with several or serious comorbidities. These more severe cases mainly present with dyspnea, reduced blood oxygen saturation, severe pneumonia, acute respiratory distress syndrome (ARDS), thus requiring prolonged advanced respiratory support, including high-flow nasal cannula (HFNC), non-invasive mechanical ventilation (NIMV), and invasive mechanical ventilation (IMV) (2, 4, 5–7). Among critically ill adult patients with COVID-19, the proportion requiring mechanical ventilation (MV) tends to exceed 70% (6). Progressive respiratory diseases caused by SARS-CoV-2 invasion and/or secondary infection will undoubtedly lead to a proportional increase in prolonged endotracheal intubation and IMV. Consequently, subsequent tracheotomy is needed in 9.65%–42% IMV patients according to the related literature (2, 4, 8–14). Adult patients with COVID-19 expected to have long and difficult weaning, or those who experience repeated weaning failure are suitable candidates for tracheotomy.

## Main text

Although the most optimal timing of tracheotomy in COVID-19 patients remains controversial to date (15, 16), tracheotomy is a necessary and important step in airway management for COVID-19 patients with prolonged endotracheal intubation, IMV, failed extubation, and ventilator dependence. Percutaneous dilated tracheotomy (PDT) had gradually replaced the open surgical tracheotomy (OST) in the intensive care unit (ICU) due to the advantages of simple equipment required and operation technology, smaller defect, bedside operation, shorter operation time and being more economical (17–22). Due to airway opening and viral aerosol exposure, operators are at high risk of occupational exposure and SARS-CoV-2 infection in the context of tracheotomy (23–25). Therefore, tracheotomy should be performed with rigorous preoperative preparation, planning and operational procedures, experienced operators, skilled cooperation and seamless communication during operation, third-level protection measures and full personal protective equipment (PPE) to avoid SARS-CoV-2 infection in clinical practice (26–29). Nevertheless, standardized third-level protection measures and bulky PPE bring huge difficulties to the implementation of tracheotomy (10).

At present, only a few studies focused on improving PDT in IMV patients with COVID-19. Therefore, the aim of this study was to propose a safer and more practical tracheotomy, namely PDT with delayed endotracheal intubation withdrawal under the guidance of bedside ultrasonography without the conventional use of flexible fiberoptic bronchoscopy (FFB) from our clinical practice, which can be used to accurately determine the optimal pre-surgical positioning, and avoid intraoperative damage of the posterior tracheal wall and occurrence of tracheoesophageal fistula (TEF). In our COVID-19 treatment center of Heilongjiang province, a total of 7 ARDS patients induced by SARS-CoV-2 infection successfully underwent the above procedure performed by the experienced operators in an ICU separate room with laminar airflow (LAF), and no serious intraoperative and postoperative complications, transmission of SARS-CoV-2, conversion from PDT to OST, revision surgery and transfusion occurred.

### Preoperative preparation

In our COVID-19 treatment center of Heilongjiang province, all patients were confirmed by detection of SARS-CoV-2 nucleic acids on oropharyngeal swabs, nasopharyngeal swabs, or lower respiratory tract specimens. Routine preoperative examinations, including whole blood cell analysis, coagulation tests, biochemical parameters, and chest x-ray or computed tomography (CT) imaging were completed in all IMV patients with COVID-19 requiring tracheotomy. This work has been reported in line with the Standards for Quality Improvement Reporting Excellence (SQUIRE) criteria.

### Personnel preparation

A COVID-19 tracheotomy team consisting of three experienced intensivists and one senior nurse was established to perform PDT with delayed endotracheal intubation withdrawal under the guidance of bedside ultrasonography. One of the intensivists provided intraoperative bedside ultrasonography support and analgesia and sedation evaluation, the remaining two performed PDT with delayed endotracheal intubation withdrawal, and the senior nurse was responsible for the administration of analgesics, sedatives and non-depolarizing muscle relaxant, as well as intraoperative collaboration. An intensivist skilled in FFB served as a backup for emergencies. Skilled cooperation and seamless communication were essential for the successful implementation of PDT with delayed endotracheal intubation withdrawal under the guidance of bedside ultrasonography.

### Equipment preparation

In addition to the consumables and equipment required for the implementation of PDT, two tracheotomy tubes of different diameters (8.0 and 7.5 for men, 7.5 and 7.0 for women) and tracheotomy supplies needed for OST were made immediately available during the procedure. An ICU separate room with LAF assigned for the operation of PDT, and the closed endotracheal suctioning system and bidirectional design heat and moisture exchanger (HME) with viral filter were conventionally provided. The HME with viral filter could protect against most viruses and bacteria. The fraction of inspired oxygen (FiO_2_) was increased to reach 100% during the procedure due to poor oxygenation function and oxygen reserve capacity among severe and critically ill patients with COVID-19. Necessary and standardized third-level protection measures and full PPE were taken during the operation, and proper and careful donning-and-doffing PPE was a prerequisite for accessing the isolation ward under the clinical supervision and guidance of full-time staff in the Infection Control Department.

### Drug preparation

1% lidocaine with 1:100,000 epinephrine was used for local anesthesia. Sedatives such as propofol and midazolam, analgesics such as remifentanil and sufentanil, and non-depolarizing muscle relaxants were administrated during the procedure. The sedation target was a Ramsay sedation score of 5 points. Non-invasive and minimally invasive hemodynamic monitoring methods were used to frequently monitor hemodynamic status in order to timely apply vasoactive drugs and correct deteriorating hemodynamics. All drugs that were expected to be used during operation and rescue were prepared in advance. Detailed and adequate preparation shortens the operation time, improves the operation safety, and reduces the risk of occupational exposure and transmission of SARS-CoV-2.

### PDT with delayed endotracheal intubation withdrawal under the guidance of bedside ultrasonography

**Figure 1:**
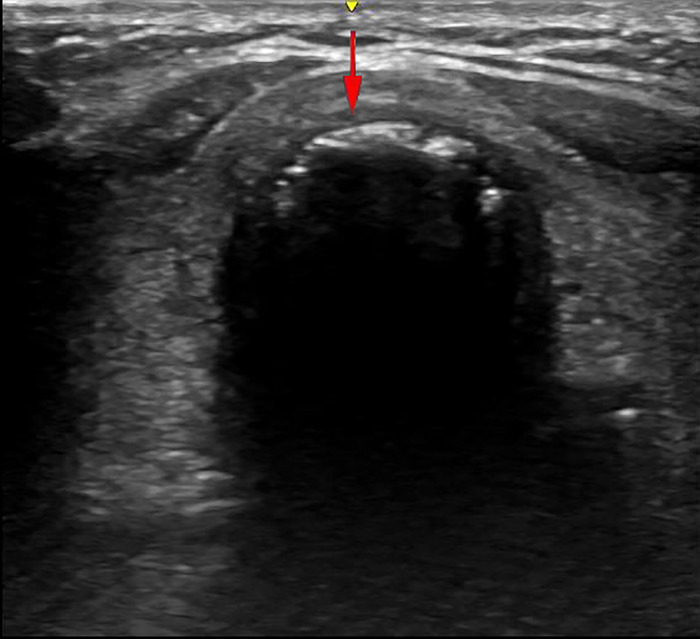
The inter-annular space between the second and third tracheal ring. The yellow and red arrows are the indication marks of the ultrasound probe and the direction of the puncture needle, respectively.

**Figure 2:**
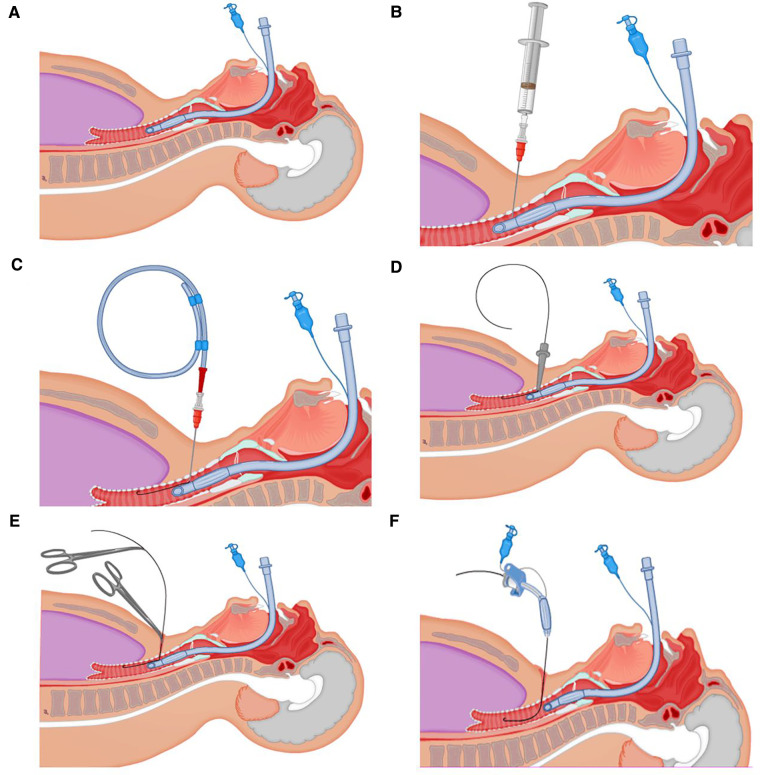
Detailed procedures. (**A**) After adequate suction of the oral cavity and supraglottic secretions, the cuff of endotracheal intubation was deflated. (**B**) The puncture cannula had entered the trachea and was located between the anterior wall of the trachea and endotracheal intubation. (**C**) The guidewire was inserted 10–15 cm along the puncture cannula. (**D**) The skin expander was rotationally inserted along the guidewire. (**E**) The endotracheal intubation remained in place until the tapered dilating forceps were withdrawn. (**F**) The tracheotomy tube with deflated cuff and inner cannula was inserted along the guidewire.

In our COVID-19 treatment center of Heilongjiang province, a disposable Portex PDT kit was used with the guide wire dilator forceps (GWDF) technique. The patient was positioned flat on the back, and shoulder support was always maintained to obtain a proper neck and head hyperextension to facilitate adequate exposure of the trachea. The optimal pre-surgical positioning was usually in the inter-annular space between the second and third tracheal ring identified by bedside ultrasonography, as shown in Figure 1. After routine disinfection and infiltration anesthesia, the skin and superficial cervical fascia were horizontally incised about 1.5–3 cm at the optimal pre-surgical positioning. After adequate suction of the oral cavity and supraglottic secretions, the cuff of endotracheal intubation was deflated, and the endotracheal intubation still remained in place, as shown in Figure 2A. After drawing 2 ml normal saline, the puncture needle with the puncture needle core (inclined face down) and the puncture cannula were vertically inserted into the trachea. After a sense of breakthrough, a large number of bubbles could be seen inside the retracted puncture needle. The puncture needle core was pulled out after the puncture needle was inserted 0.5 cm more. At this point, the syringe was directly connected to the puncture cannula. If a large number of bubbles were still visible inside the retracted syringe, it was confirmed that the puncture cannula had entered the trachea and was located between the anterior wall of the trachea and endotracheal intubation, as shown in Figure 2B. The guidewire was inserted 10–15 cm along the puncture cannula, as shown in Figure 2C. After pulling out the puncture cannula, the skin expander was rotationally inserted along the guidewire, as shown in Figure 2D. The subcutaneous tissue, muscular layer, and anterior wall of the trachea were expanded with the tapered dilating forceps along the guidewire in turn until the tracheotomy tube could be accommodated. The endotracheal intubation always remained in place until the tapered dilating forceps were withdrawn, as shown in Figure 2E, which has a protective role on the posterior wall of the trachea, thus avoiding the injury to the posterior wall of the trachea and the occurrence of TEF. After endotracheal intubation was retracted to 14–16 cm away from the incisor (16 cm for men and 14 cm for women), the tracheotomy tube with deflated cuff and inner cannula was inserted along the guidewire (Figure 2F), after which the guidewire and inner cannula were pulled out. The correct positioning of the tracheotomy tube was confirmed through end-tidal carbon dioxide partial pressure monitoring and was securely fixed after the cuff was inflated, after which IMV was resumed. The endotracheal intubation was completely pulled out, and the incision was covered with gauze.

### Postoperative management

Upon the completion of the operation, reusable medical devices exposed to COVID-19 patients were disinfected according to the national and hospital disinfection protocol for SARS-CoV-2. Disposable equipment and the waste were properly disposed of in a standard way. Bilateral lung auscultation and chest x-ray or CT imaging were routinely performed after the operation. The gauze at the tracheotomy tube was changed daily to allow early detection of complications such as bleeding and local infection, and the tightness of neck fixation was monitored in order to prevent tracheotomy tube prolapse. Postoperative minimal bleeding, as the most common operative complication in patients with PDT, did not require revision surgery and transfusion after medical therapy or conservative remedies or both, such as hemostatic drugs and local compression of oil gauze. The cuff pressure of the tracheotomy tube was maintained between 25 and 30 cm H_2_O and periodically monitored to keep the ventilation system closed-circuit and to prevent adverse events caused by the overinflation of the cuff.

## Discussion

The COVID-19 global pandemic has gradually affected every aspect of patients’ care, including appointment, triage, visiting, attending, treatment strategy and operation, which were often left with little guidance. Experience on improved PDT in IMV patients with COVID-19 is lacking (30, 31). Although practices with non-COVID-19 ARDS patients can serve as a useful reference, differences caused by unique organizational, environmental, and ethical singularities of COVID-19 are inevitable (31). Poor lung function, coagulation defect, unsustainable blood oxygen saturation, abundant thick airway secretions, and transmission of SARS-CoV-2 increase the tracheotomy-related risks, resulting in the high requirement for ICU. Timely, safe and efficient implementation of tracheotomy under the premise of ensuring the safety of the operators is a very important clinical practical problem in the management of severe and critically ill patients with COVID-19 (32).

The sequelae of prolonged endotracheal intubation are well known and recognized. Performing a tracheotomy may improve patients' comfort, lower frequency of oral lesions, clear airway secretions, reduce the dose and duration of analgesia and sedative drugs, avoid complications related to prolonged analgesia and sedation, reduce dead-space ventilation and airway resistive load, improve expiratory flow, expedite rehabilitation and allow for easier, safer and faster weaning from ventilator (17, 33–37). In clinical practice, the decision to perform tracheotomy should be based on the balance between exact expected benefits and optimal care of the selected patients and the possible risk of SARS-CoV-2 transmission to operators. The selection of the appropriate tracheotomy techniques for COVID-19 patients, i.e., OST or PDT, is usually determined based on the patient's overall clinical condition, local expertise and training, operating experience, resource utilization, and operators preference, as the overall complication rate is similar for both (23, 38–40). At present, PDT performed by intensivists has become the mainstream tracheotomy technique in ICU.

PDT is usually performed by intensivists at the bedside and guided by FFB or bedside ultrasound instead of transferring patients to the operating room, which can be cost-effective, and minimize the risk of viral transmission and disease progression in transit (41, 42). However, in PDT, the ventilator circuit needs to be opened more often and produces more viral aerosols are produced (8), thus increasing the risk of exposure due to more extensive airway manipulation (42), especially in the case of FFB guidance. FFB-guided PDT also needs more operators and patients expenses, prolonged operation time, increased frequency of airway obstruction, hypercapnia, and coughing reflex (43), all of which should be avoided during tracheotomy. In addition, intraoperative bleeding and abundant thick airway secretions can seriously affect the guiding effect of FFB. Therefore, FFB-guided PDT is not appropriate for emergency PDT and patients with upper airway obstruction, severe ventilation and air exchange dysfunction. However, in the absence of FFB guidance, there is a certain degree of blindness in the process of blind puncture of the puncture needle and expansion of the tapered dilating forceps, which can easily increase the incidence of complications, especially the intraoperative damage of the posterior tracheal wall and the occurrence of TEF. TEF has been regarded as a rare but life-threatening complication after tracheotomy, with an incidence of 1% approximately (44). Therefore, in our clinical practice, we adjusted the standard PDT operation procedure. Under the premise of unconventional use of FFB guidance, the timing of endotracheal intubation withdrawal is delayed, i.e., endotracheal intubation remains in place in the process of blind puncture of the puncture needle and expansion of the tapered dilating forceps, which has a protective role on the posterior wall of the trachea, thus avoiding the injury to the posterior wall of the trachea and the occurrence of TEF. The average airway diameters in normal males and females were 1.2–1.5 and 1.0–1.3 cm, respectively, while the inner diameters of 7.0, 7.5, and 8.0 endotracheal intubation were 7.0, 7.5, and 8.0 mm, respectively, which indicated there was enough space between the anterior wall of the trachea and endotracheal intubation to perform the above operation procedures when the cuff of endotracheal intubation was not inflated (Figures 2B–F).

Bedside ultrasonography has an important role in overcoming operational difficulties caused by standardized third-level protection measures and bulky PPE, especially in determining the optimal pre-surgical positioning for COVID-19 patients with ambiguous surface position, obesity, short neck, or limited neck extension. Due to rich blood vessels and common vascular anatomical variations in the neck region, bedside ultrasonography is recommended to be systematically performed before tracheotomy to ensure no large vessel or thyroid isthmus in the puncture area in order to prevent intraoperative tissue damage and postoperative bleeding. Preoperative bedside ultrasonography of the neck region with visualization of large blood vessels, thyroid, and trachea are critical to accurately understand anatomy of the neck region and safely implement tracheotomy in IMV patients with COVID-19. Therefore, bedside ultrasonography has a broad application prospect in ICU, especially for critically ill COVID-19 patients with hypoxemia and hemodynamic failure who are unable to tolerate transport (45).

Due to the generation of aerosols with high viral loads, it is necessary to ensure the safety of operators and avoid the transmission of SARS-CoV-2 in the process of PDT (46). Based on our clinical experiences and related literature, appropriate and sufficient infection control measures should be taken as follows. First, ideally, PDT should be performed until at least 1 day apart after consecutive negative detection of SARS-CoV-2 nucleic acids from the lower respiratory tract specimens among ARDS patients with COVID-19 (47). This period means the acute phase of SARS-CoV-2 infection to pass, viral load to decrease, and the risk of transmission to lower. If available, the test of SARS-CoV-2 viral load in the airway secretion can be used as a reasonable surrogate for viral clearance and an accurate indicator of PDT timing, with a cycle threshold (CT) value close to 40 indicating a low risk of transmission in the context of tracheotomy (48). This is particularly important in severe and critically ill patients with COVID-19, who have a higher SARS-CoV-2 viral load and slower descent than mild patients with COVID-19 (49). Second, ideal protection for operators is achieved when PDT is performed under deep sedation and muscle relaxation to inhibit coughing reflex, since the spread of viral aerosols after cough is practically unstoppable, reaching up to 3.6 km/h (2.25 miles/h), and viral aerosols are almost vertically exposed towards the facial area of operators in less than a second within the range of 7–8 m (18, 50). Third, prior to opening the anterior wall of the trachea, IMV should be suspended for a short time to reduce the production of viral aerosols, if tolerated (51, 52). IMV can be resumed after the tracheotomy tube is confirmed in place and the cuff is properly inflated. Certainly, increasing FiO_2_ to reach 100% for adequate pre-oxygenation to enhance respiratory reserve is also a very important step before that. Fourth, in our COVID-19 treatment center of Heilongjiang province, PDT has been performed bedside in an ICU separate room with LAF in order to greatly dilute the virus-containing aerosol concentration, and reduce the contamination, thus lowering the risk of SARS-CoV-2 transmission (18). Fifth, if IMV patients with COVID-19 are routinely receiving pharmacologic anticoagulation, it must be stopped for more than 24 h before PDT to avoid the increased risk of bleeding and oozing. Last, the minimal-staff policy should be followed for each PDT, i.e., all medical staff not related to the operation of PDT should not be on site.

PDT with delayed endotracheal intubation withdrawal was first proposed by our team, after which it has been successfully implemented for many years in clinic, with good clinical results (53, 54). Although PDT with delayed endotracheal intubation withdrawal under the guidance of bedside ultrasonography has only been successfully performed in 7 ARDS patients induced by SARS-CoV-2 infection in our COVID-19 treatment center of Heilongjiang province, it is both innovative and illuminating.

## Conclusion

Based on our clinical practice, in this study, we introduced a safer and more practical tracheotomy, i.e., PDT with delayed endotracheal intubation withdrawal under the guidance of bedside ultrasonography without the conventional use of FFB, which can be conducive to accurately determine the optimal pre-surgical positioning and avoid intraoperative damage of the posterior tracheal wall and the occurrence of TEF, thus having important innovative and practical significance that should be promoted in clinical practice.

## Data Availability

The original contributions presented in the study are included in the article/Supplementary Material, further inquiries can be directed to the corresponding authors.
